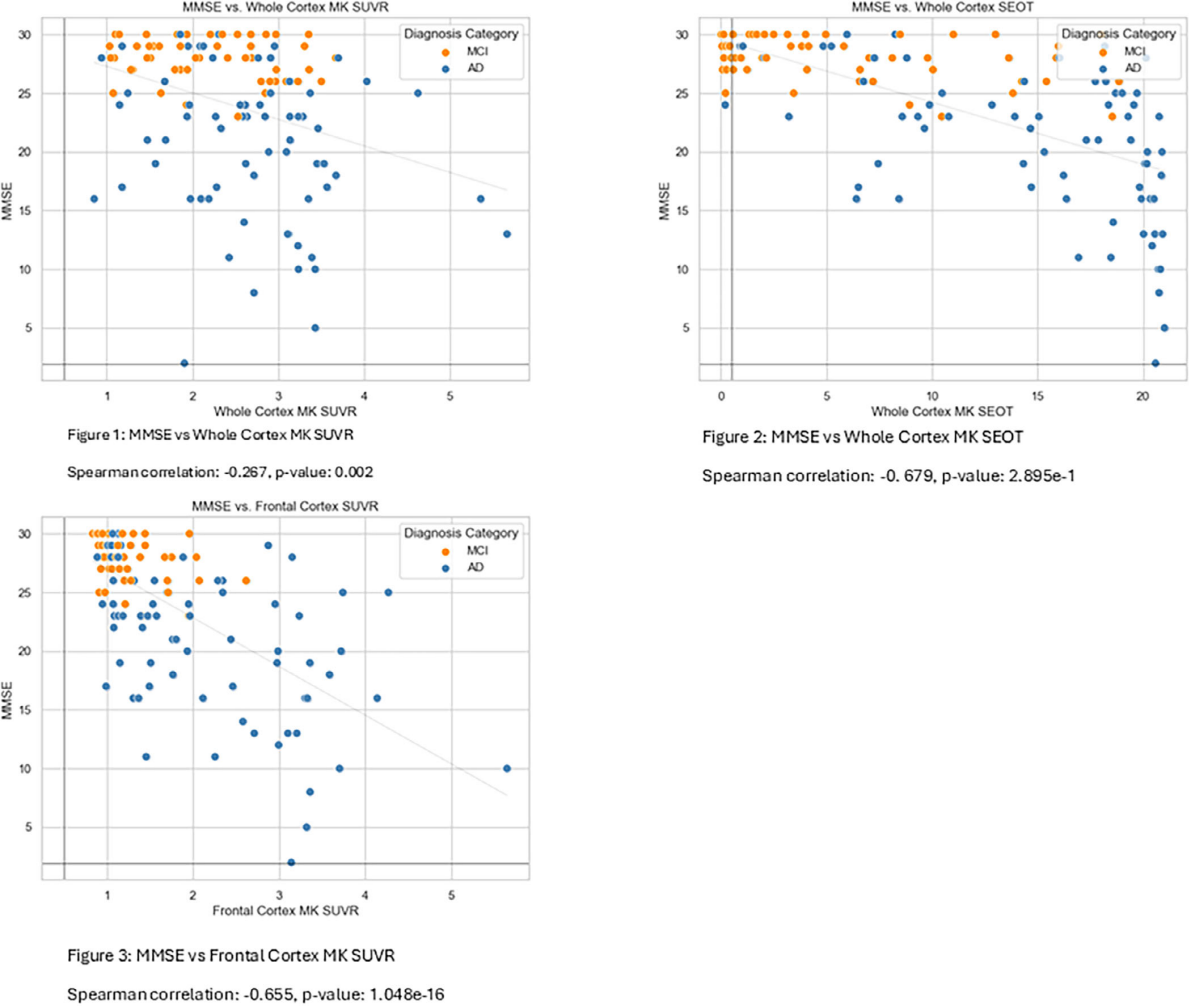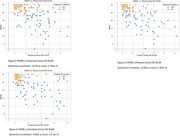# Associations between brain and cognitive resilience, tau load and extent in Alzheimer's disease

**DOI:** 10.1002/alz70856_107705

**Published:** 2026-01-09

**Authors:** Stuart William Mitchell, Tevy Chan, Lydia Trudel, Seyyed Ali Hosseini, Arthur C. Macedo, Marina P Gonçalves, Nesrine Rahmouni, Brandon J Hall, Kely Monica Quispialaya Socualaya, Joseph Therriault, Stijn Servaes, Gleb Bezgin, Yansheng Zheng, Etienne Aumont, Yi‐Ting Wang, Jaime Fernandez Arias, Ana Paula Bernardes Real, Wan Lu Jia, Robert Hopewell, Chris Hsiao, Jean‐Paul Soucy, Paolo Vitali, Tharick A Pascoal, Pedro Rosa‐Neto

**Affiliations:** ^1^ Translational Neuroimaging Laboratory, The McGill University Research Centre for Studies in Aging, Montréal, QC, Canada; ^2^ McGill University, Montreal, QC, Canada; ^3^ Montreal Neurological Institute, Montreal, QC, Canada; ^4^ McGill University Research Centre for Studies in Aging, Montreal, QC, Canada; ^5^ The McGill University Research Centre for Studies in Aging, Montreal, QC, Canada; ^6^ Translational Neuroimaging Laboratory, The McGill University Research Centre for Studies in Aging, Montreal, QC, Canada; ^7^ Translational Neuroimaging Laboratory, Montréal, QC, Canada; ^8^ 6630 Sherbrooke West, app. 1605, Montréal, QC, H4B 1N7, Montréal, QC, Canada; ^9^ McGill University, Montréal, QC, Canada; ^10^ LR12SP11 Sahloul University Hospital, Sousse, Tunisia; ^11^ Imaging Genetics Center, Mark and Mary Stevens Neuroimaging and Informatics Institute, Keck School of Medicine, University of Southern California, Marina del Rey, CA, USA; ^12^ McGill Centre for Studies in Aging, Department of Neurology and Neurosurgery, McGill University, Montreal, QC, Canada; ^13^ Montreal Neurological Institute, Montréal, QC, Canada; ^14^ Université du Québec à Montréal, Montréal, QC, Canada; ^15^ Translational Neuroimaging Laboratory / Montreal Neurological Institute, Montreal, QC, Canada; ^16^ Jenny de Andrade Faria Institute – Reference Center for the Elderly, Federal University of Minas Gerais (UFMG), Belo Horizonte, Brazil, Belo Horizonte, Minas Gerais, Brazil; ^17^ Sciences Applied to Adult Health Postgraduate Program, Faculty of Medicine, Universidade Federal de Minas Gerais (UFMG), Belo Horizonte, Minas Gerais, Brazil; ^18^ Department of Geriatrics, McGill University, Montreal, QC, Canada; ^19^ Hospital das Clínicas da UFMG, University Hospital, Universidade Federal de Minas Gerais (UFMG), Belo Horizonte, Minas Gerais, Brazil; ^20^ USP, São Paulo, São Paulo, Brazil; ^21^ Cog‐Aging Research Group, Universidade Federal de Minas Gerais (UFMG), Belo Horizonte, Minas Gerais, Brazil; ^22^ Jewish General Hospital, Montreal, QC, Canada; ^23^ McGill University Centre for Studies in Aging, Montreal, QC, Canada; ^24^ Division of Geriatric Medicine, McGill University, Montreal, QC, Canada; ^25^ Goldman Herzl Family Practice Centre, Montreal, QC, Canada; ^26^ Department of Neurology and Neurosurgery, McGill University, Montreal, QC, Canada; ^27^ Department of Family Medicine, McGill University, Montreal, QC, Canada; ^28^ Douglas Mental Health University Institute, Montreal, QC, Canada; ^29^ PERFORM Centre ‐ Concordia University, Montréal, QC, Canada; ^30^ PERFORM Centre, Concordia University, Montreal, QC, Canada; ^31^ Université de Montréal, Montréal, QC, Canada; ^32^ Montreal Neurological Institute, McGill University, Montréal, QC, Canada; ^33^ University of Montreal hospital centre, Montreal, QC, Canada; ^34^ McConnell Brain Imaging Centre, Montreal Neurological Institute and Hospital, McGill University, Montreal, QC, Canada; ^35^ Montreal Neurological Institute, McGill University, Montreal, QC, Canada; ^36^ Translational Neuroimaging, Montréal, QC, Canada; ^37^ Centre hospitalier de l’Université de Montréal, Montréal, QC, Canada; ^38^ CIUSSS du Nord‐de‐l'Île‐de‐Montréal, Montreal, QC, Canada; ^39^ University of Pittsburgh, Pittsburgh, PA, USA; ^40^ Translational Neuroimaging Lab, Verdun, QC, Canada; ^41^ McGill University Research Centre for Studies in Aging, Verdun, QC, Canada; ^42^ Department of Psychiatry and Neurology, Pittsburgh, PA, USA; ^43^ Douglas Hospital Research Centre, Verdun, QC, Canada; ^44^ University of Pittsburgh School of Medicine, Pittsburgh, PA, USA; ^45^ Departments of Psychiatry and Neurology, University of Pittsburgh School of Medicine, Pittsburgh, PA, USA; ^46^ Centre for Studies on Prevention of Alzheimer's Disease (StoP‐AD Centre), Douglas Mental Health Institute, Verdun, QC, Canada; ^47^ McConnell Brain Imaging Centre ‐ McGill University, Montreal, QC, Canada; ^48^ McGill Center for Research Studies in Aging, Montréal, QC, Canada; ^49^ 6825 LaSalle Boulevard, Montreal, QC, Canada; ^50^ McGill Centre for Studies in Aging, Montreal, QC, Canada; ^51^ McGill Centre for Studies in Aging, Alzheimer's Disease Research Unit, Montreal, QC, Canada; ^52^ Translational Neuroimaging Laboratory, Montreal, QC, Canada; ^53^ Centre for Studies on Prevention of Alzheimer's disease (StoP‐AD Centre), Montreal, QC, Canada; ^54^ McGill University Research Centre for Studies in Aging, Douglas Research Centre, Montreal, QC, Canada; ^55^ McConnell Brain Imaging Centre, Montreal Neurological Institute, McGill University, Montreal, QC, Canada; ^56^ Department of Neurology and Neurosurgery, McGill University, Montréal, QC, Canada; ^57^ Department of Psychiatry, McGill University, Montreal, QC, Canada

## Abstract

**Background:**

Brain and cognitive resilience (BR, CR) reflect the capacity to maintain structural integrity and cognitive function despite pathological tau deposition in Alzheimer's disease (AD). Tau pathology can be characterized in terms of spatial extent of tauopathy (SEOT) or load using standardized uptake value ratio (SUVR). The aim was to compare SEOT and SUVR in their association with BR and CR. To replicate findings from Ossenkoppele et al. (2020) using MK‐6240 PET imaging and evaluate demographic, genetic, and imaging factors associated with BR and CR. The objective of this study is to assess the value of SEOT metrics in resilience models and compare their predictive power to standardized uptake value ratio (SUVR) and to evaluate cross sectional interactions between tau pathology, cognitive resilience, and cognitive decline.

**Method:**

We assessed 126 amyloid‐β‐positive participants TRIAD cohort with tau‐PET using [^18^F]MK6240 and cognitive assessments (MMSE). SEOT was quantified as the proportion of voxels considered as abnormal relative to young controls. We used Participants recruited from TRIAD cohort, including individuals with mild cognitive impairment (MCI) or AD, positive amyloid‐β biomarkers, MK‐6240 PET imaging data.

**Result:**

Higher Whole Cortex MK SUVR is associated with lower MMSE scores, showing increased tau pathology correlates with cognitive decline. MCI patients maintain higher MMSE scores despite some tau accumulation, while AD patients show greater variability and decline. The negative trend suggests tau deposition contributes to cognitive impairment, but other factors may also play a role.

2. Whole Cortex MK SUVR vs. MMSE the negative correlation between Whole Cortex SEOT and MMSE appears stronger, with a more pronounced decline in cognitive function (MMSE scores) as SEOT increases, suggesting SEOT may be a more sensitive marker of disease progression in AD patients.

**Conclusion:**

Whole Cortex SEOT exhibits a stronger negative correlation with MMSE compared to Whole Cortex MK‐6240 SUVR, indicating that SEOT may serve as a more sensitive marker of cognitive decline in Alzheimer's disease and mild cognitive impairment. Further research is needed to validate SEOT's potential as a diagnostic or prognostic biomarker in neurodegenerative conditions.